# Supplementary material for: Empirical methods for controlling false positives and estimating confidence in ChIP-Seq peaks
Source: BMC Bioinformatics. 2008 Dec 5;9:523. doi: 10.1186/1471-2105-9-523 (PMC2628906; doi:10.1186/1471-2105-9-523)
Supplement: Additional File 1 — A variety of html documents from the USeq web site detailing the available applications, their best usage, output file type descriptions, command line menus, etc. [file 1471-2105-9-523-S1.zip › USeqUserGuides/JavaDoc.zip!/JavaDoc/selex/SelexParams.html]

SelexParams


|  |  |  |
| --- | --- | --- |
| **Overview** \| **Package** \| **Class** \| **Use** \| **Tree** \| **Deprecated** \| **Index** \| **Help** | |  |
| PREV CLASS   **NEXT CLASS** | **FRAMES**    **NO FRAMES**     **All Classes** |  |
| SUMMARY: NESTED \| FIELD \| CONSTR \| METHOD | DETAIL: FIELD \| CONSTR \| METHOD |  |


---


## selex Class SelexParams

```
java.lang.Object
  selex.SelexParams
```

---

``` public class SelexParams extends java.lang.Object ```

Hold parameters and results generated by the `SelexSeqParser`; also generates reports.

---

**Constructor Summary**

`SelexParams()`


| **Method Summary** | |
| --- | --- |
| `void` | `addExpectedSubSeq(int expects)` |
| `void` | `addObservedSubSeq(int obs)` |
| `void` | `addObsExpRatio(double ratio)` |
| `void` | `appendLength(int x)` |
| `void` | `appendSub(java.lang.String sub)` |
| `int` | `getAveExpSeqLength()` |
| `int` | `getAveNumExpSubSeqs()` |
| `int` | `getAveNumObsSubSeqs()` |
| `java.lang.String` | `getAveObsExpRatio()` |
| `int` | `getExpectedSize()` |
| `java.lang.String` | `getLeftSideMatch()` |
| `java.lang.String` | `getLeftSideReplace()` |
| `int` | `getLenMax()` |
| `int` | `getLenMin()` |
| `java.lang.String` | `getQualFile(int index)` |
| `java.lang.String[]` | `getQualFiles()` |
| `java.lang.String` | `getRestSite()` |
| `java.lang.String` | `getRightSideMatch()` |
| `java.lang.String` | `getRightSideReplace()` |
| `java.lang.StringBuffer` | `getRunning()` |
| `int` | `getScoreCutOff()` |
| `java.lang.String` | `getSeqFile(int index)` |
| `java.lang.String[]` | `getSeqFiles()` |
| `void` | `incNumFiles(int x)` |
| `void` | `incNumSeqReads(int x)` |
| `void` | `incNumSubs(int x, int y, int z)` |
| `void` | `incrementNumBadSeqReads()` |
| `void` | `incrementNumNoInsertSeqReads()` |
| `void` | `incrementNumShortInsertSeqReads()` |
| `java.lang.String` | `makeFinalReport()` |
| `boolean` | `modifyEnds()` |
| `java.lang.String` | `plotHistogram()` |
| `void` | `printSave(java.lang.String newString)` |
| `void` | `processArgs(java.lang.String[] args)` |
| `void` | `writeReport()` |
| `void` | `writeSubSeqFasta()` |

**Methods inherited from class java.lang.Object**

`equals, getClass, hashCode, notify, notifyAll, toString, wait, wait, wait`

**Constructor Detail**

### SelexParams

```
public SelexParams()
```


**Method Detail**

### getLeftSideMatch

```
public java.lang.String getLeftSideMatch()
```

---


### getLeftSideReplace

```
public java.lang.String getLeftSideReplace()
```

---


### modifyEnds

```
public boolean modifyEnds()
```

---


### getRightSideMatch

```
public java.lang.String getRightSideMatch()
```

---


### getRightSideReplace

```
public java.lang.String getRightSideReplace()
```

---


### getRestSite

```
public java.lang.String getRestSite()
```

---


### getSeqFile

```
public java.lang.String getSeqFile(int index)
```

---


### getSeqFiles

```
public java.lang.String[] getSeqFiles()
```

---


### getQualFile

```
public java.lang.String getQualFile(int index)
```

---


### getQualFiles

```
public java.lang.String[] getQualFiles()
```

---


### getScoreCutOff

```
public int getScoreCutOff()
```

---


### getLenMin

```
public int getLenMin()
```

---


### getLenMax

```
public int getLenMax()
```

---


### getExpectedSize

```
public int getExpectedSize()
```

---


### getAveNumExpSubSeqs

```
public int getAveNumExpSubSeqs()
```

---


### getAveNumObsSubSeqs

```
public int getAveNumObsSubSeqs()
```

---


### getAveExpSeqLength

```
public int getAveExpSeqLength()
```

---


### getAveObsExpRatio

```
public java.lang.String getAveObsExpRatio()
```

---


### getRunning

```
public java.lang.StringBuffer getRunning()
```

---


### addObsExpRatio

```
public void addObsExpRatio(double ratio)
```

---


### addExpectedSubSeq

```
public void addExpectedSubSeq(int expects)
```

---


### addObservedSubSeq

```
public void addObservedSubSeq(int obs)
```

---


### incrementNumShortInsertSeqReads

```
public void incrementNumShortInsertSeqReads()
```

---


### incrementNumNoInsertSeqReads

```
public void incrementNumNoInsertSeqReads()
```

---


### incrementNumBadSeqReads

```
public void incrementNumBadSeqReads()
```

---


### processArgs

```
public void processArgs(java.lang.String[] args)
```

---


### printSave

```
public void printSave(java.lang.String newString)
```

---


### appendSub

```
public void appendSub(java.lang.String sub)
```

---


### incNumSubs

```
public void incNumSubs(int x,
                       int y,
                       int z)
```

---


### appendLength

```
public void appendLength(int x)
```

---


### incNumFiles

```
public void incNumFiles(int x)
```

---


### incNumSeqReads

```
public void incNumSeqReads(int x)
```

---


### makeFinalReport

```
public java.lang.String makeFinalReport()
```

---


### writeSubSeqFasta

```
public void writeSubSeqFasta()
```

---


### writeReport

```
public void writeReport()
```

---


### plotHistogram

```
public java.lang.String plotHistogram()
```


---


|  |  |  |
| --- | --- | --- |
| **Overview** \| **Package** \| **Class** \| **Use** \| **Tree** \| **Deprecated** \| **Index** \| **Help** | |  |
| PREV CLASS   **NEXT CLASS** | **FRAMES**    **NO FRAMES**     **All Classes** |  |
| SUMMARY: NESTED \| FIELD \| CONSTR \| METHOD | DETAIL: FIELD \| CONSTR \| METHOD |  |


---
